# No difference in postprandial mesenteric blood flow between healthy younger and elderly individuals

**DOI:** 10.1038/s41598-024-58111-w

**Published:** 2024-04-15

**Authors:** Thomas Hartwig Siebner, Jens Dahlgaard Hove, Christopher Fugl Madelung, Oliver James Hulme, Flemming Bendtsen, Hartwig Roman Siebner, Mads Barløse

**Affiliations:** 1 Danish Research Centre for Magnetic Resonance, Centre for Functional and Diagnostic Imaging and Research, Copenhagen University Hospital - Amager and Hvidovre, Copenhagen, Denmark; 2 Department of Cardiology, Copenhagen University Hospital - Amager and Hvidovre, Copenhagen, Denmark; 3 Department of Clinical Medicine, Faculty of Health and Medical Sciences, University of Copenhagen, Copenhagen, Denmark; 4 Department of Neurology, Copenhagen University Hospital - Bispebjerg and Frederiksberg, Copenhagen, Denmark; 5 Department of Psychology, Faculty of Social Sciences, University of Copenhagen, Copenhagen, Denmark; 6 Gastrounit, Medical Division, Copenhagen University Hospital - Amager and Hvidovre, Copenhagen, Denmark; 7 Department of Clinical Physiology and Nuclear Medicine, Centre for Functional and Diagnostic Imaging and Research, Copenhagen University Hospital - Amager and Hvidovre, Copenhagen, Denmark

**Keywords:** Physiology, Gastroenterology

## Abstract

We recently used phase-contrast magnetic resonance imaging (PC-MRI) to demonstrate an attenuated postprandial blood flow response in the superior mesenteric artery (SMA) in patients with Parkinson's disease compared to age- and sex-matched healthy controls. Since both groups showed substantial inter-individual variations, we extended the cohort of controls with a group of young individuals to investigate possible age-related effects. Seventeen healthy young subjects aged < 30 years and 17 elderly subjects aged > 50 years underwent serial PC-MRI to measure the postprandial blood flow response in the SMA after ingestion of a standardized liquid test meal (∼400 kcal). Postprandial blood flow dynamics in SMA did not differ between young and elderly subjects. A noticeable inter-individual variation in postprandial intestinal blood flow increase was found, and approximately 30% of the variation could be explained by the preprandial blood flow. Regardless of age, some subjects showed a remarkable transient SMA blood flow increase immediately after meal intake. This study provides tentative evidence that postprandial blood flow dynamics in SMA in healthy young and elderly subjects do not substantially differ, indicating that age is without impact on vascular response in SMA as an indicator for regulation of mesenteric perfusion in response to food intake.

## Introduction

Ingestion and digestion of food triggers a substantial increase in gastrointestinal regional blood flow which parallels an increase in motor, secretory and absorptive activities of the splanchnic organs^1–3^. Postprandial hyperemia secures the supply of essential nutrients and oxygen to the splanchnic organs to optimize digestion and facilitate absorption and transport of digested substances to the liver^1,3,4^.

Following a meal intake the ingested food mixes with gastric juices in the stomach to become chyme and is afterwards slowly emptied into the duodenum, where it is further digested as it travels through the small intestine^2^. Earlier studies suggested that blood flow in the arteries supplying the gastrointestinal tract, including the celiac artery, superior mesenteric artery (SMA) and inferior mesenteric artery, increases sequentially as the digested chyme is transported along the gastrointestinal tract and is exposed to the mucosal surface supplied by each particular artery^5–9^. Following nutrient absorption, the blood flow to each segment returns to baseline after the digestive chyme has passed the region of the digestive tract. In line with this, it has been shown, that subjects fed intraluminally, i.e. orally and enterally, demonstrate significant postprandial increase in SMA blood flow, while hemodynamically stable patients fed parenterally show decreased postprandial SMA blood flow^10^. In addition, intraarterial injection of glucose did not seem to alter microvascular intestinal blood flow when using exteriorized segments of jejunum from dogs^11^, suggesting that the endoluminal exposure to glucose is a prerequisite for gastrointestinal blood flow increase. However, Someya et al. showed an increase in mean blood velocity in SMA within a minute after start of the meal, suggesting that the increase in SMA blood flow might precede the arrival of the chyme to the small intestine^3^.

The local postprandial hyperemic response also depends on the composition of the meal^3,4,8,9,12,13^. The ingestion of glucose but not water, saline or lactulose solutions triggers a blood flow increase in SMA in healthy individuals^14–17^. Furthermore, healthy individuals display a positive dose–response relationship between the ingested meal energy content and the postprandial blood flow response in the SMA^18,19^.

Recently, we introduced a novel method to study postprandial blood flow regulation using phase-contrast magnetic resonance imaging (PC-MRI) and applied the method in patients with Parkinson’s disease^20^. Patients with Parkinson’s disease showed an attenuated postprandial blood flow response in the SMA in response to oral intake of a standardized liquid test meal compared to healthy individuals^20^. Both, patients with Parkinson’s disease and the age- and sex-matched healthy controls with comparable body mass, showed substantial inter-individual variations in postprandial intestinal blood flow increase. We explored the possibility that differences in age might have contributed to this inter-individual variability in a post-hoc analysis. The subject’s age correlated neither with SMA blood flow values at pre-ingestion baseline and postprandial maximum nor with the absolute blood flow increase. Since the age range of the study cohort was limited, this post-hoc finding was inconclusive. We therefore decided to extend the preexisting cohort of healthy controls with a group of healthy young individuals to test for a possible difference in postprandial blood flow regulation with age. This enabled us to investigate whether blood flow dynamics in the SMA in response to meal ingestion differs between healthy young and elderly subjects.

## Results

Demographic and clinical group data along with the statistical results of between-group comparisons are listed in Table 1. All participants completed the study without reporting any adverse events. Mean body mass index of the elderly group was within the overweight range and significantly higher compared to the young group (*p* = 0.008, Table 1). There was no difference in the reported amount of upper gastrointestinal symptoms. Fasting levels of blood glucose were normal (< 6.3 mmol/l) in all participants.

**Table 1:** Demographic and clinical data of healthy young and elderly subjects.

	Young (n = 17)	Elderly (n = 17)	P-value
Gender (male/female)	11/6	11/6	
Age (years)	25 (20–27)	57 (52–66)	< 0.0001
Body mass index (kg/m^2^)	23.2 ± 3.3	26.4 ± 3.3	0.008
**GSRS (Items 1–9)**			
Total score	1.22 (1–1.53)	1.22 (1–1.71)	0.55
**Superior mesenteric artery blood flow (BF) (l/min)**			
Baseline BF	0.41 ± 0.12	0.41 ± 0.14	1
Postprandial maximal BF	1.1 ± 0.31	1.11 ± 0.28	0.91
Postprandial increase in BF	0.69 ± 0.27	0.7 ± 0.22	0.89
Time to maximum (min)	28.8 (23.8–43)	30.2 (18.1–43.2)	0.95
Smoothed postprandial BF changes*^1^			
Relative increase in BF (%)	181 ± 64	195 ± 85	0.59
Time to maximum (min)	28.7 (23.1–43.1)	29.7 (18.3–42.9)	0.92
Maximal slope (l/min)	0.07 (0.04–0.13)	0.07 (0.05–0.09)	0.92
**Blood glucose (BG) (mmol/l)**			
Baseline BG	4.8 ± 0.41	4.7 ± 0.44	0.69
Postprandial maximal BG	7.5 ± 0.9	7.3 ± 0.9	0.55
Postprandial increase in BG	2.7 ± 0.7	2.6 ± 0.8	0.64
Time to maximum (min)	51.1 ± 10	55.9 ± 10.5	0.18

Data given as mean ± SD or median (10% quantile**–**90% quantile).

*^1^A local polynomial regression fitting curve was fitted to the individual time series of blood flow measurements applying a 25% smoothing span, and then blood flow estimates were predicted at a sample rate of one sample per minute.

### Postprandial blood flow and blood glucose changes

**Figure 1:**
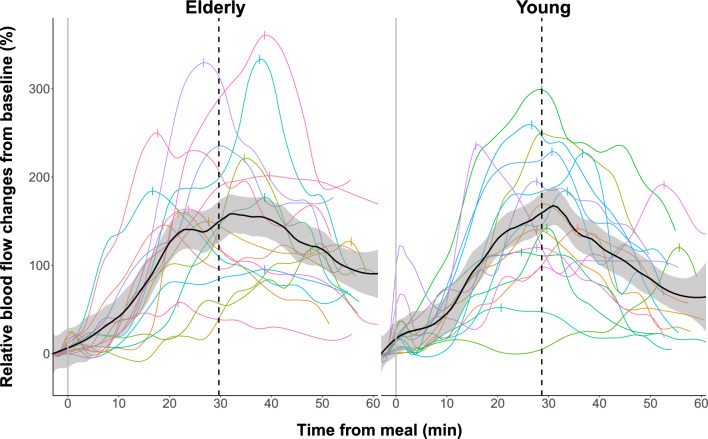
Relative smoothed postprandial superior mesenteric artery blood flow changes in elderly and young participants. Relative blood flow changes from baseline. Curves are fitted to the individual time series of blood flow measurements using local polynomial regression fitting with a 25% smoothing span. The black curve with shaded areas marks the mean and standard error for the elderly group and the young group, while the vertical dotted line marks the median for the maximal blood flow measurements of each group. The vertical dashes mark the maximal blood flow for each subject.

**Figure 2:**
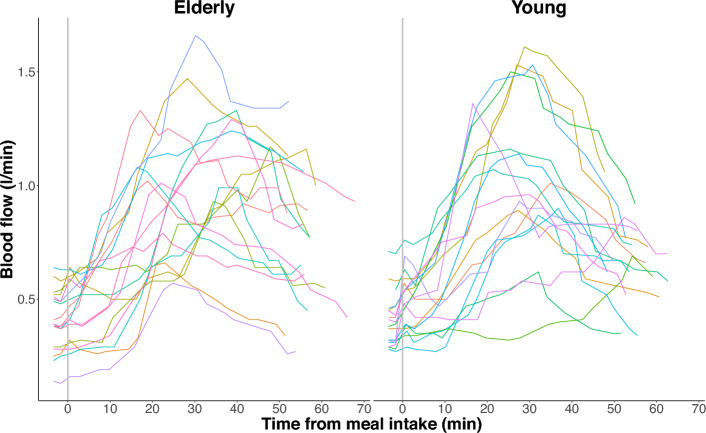
Postprandial superior mesenteric artery blood flow measurements in elderly and young participants. The absolute blood flow measurements are connected using linear interpolation.

**Figure 3:**
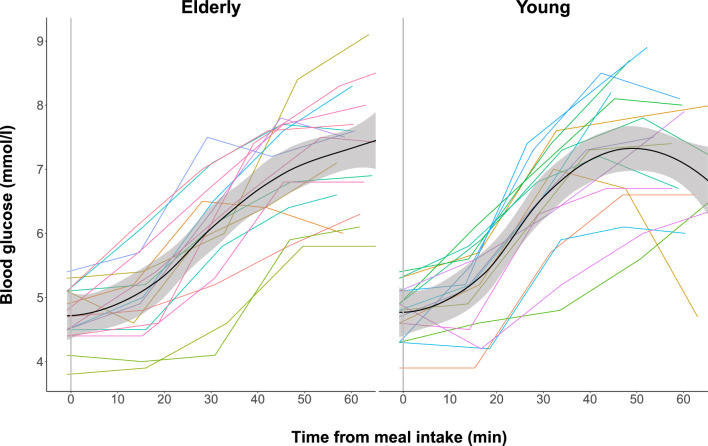
Postprandial blood glucose measurements in elderly and young participants. The absolute blood glucose measurements are connected using linear interpolation. The black curve with shaded areas marks the mean and standard error for the elderly group and the young group.

Mean group data as well as individual data are presented separately for young and elderly participants in Figs. 1, 2 and 3. All participants showed a rise in SMA blood flow and blood glucose levels after ingestion of the standardized test meal, but responses varied substantially among participants (Figs. 1, 2 and 3). At the group level, the analysis of absolute postprandial SMA blood flow increase showed no significant difference between young and elderly subjects (Two-sample t-test, *p* = 0.89, 95% CI [− 0.16, 0.18], Table 1, Fig. 1).

To draw inferences about the null hypothesis of no difference between the groups, we performed a complementary Bayesian two-sample t-test (Cauchy prior, scale factor sqrt(2)), revealing moderate evidence for the null hypothesis. The observed Bayes Factor of 3.019 indicates that the data are approximately three times more likely under the null hypothesis than the alternative. This was robust to differences in prior widths on the effect size. However, the credibility intervals suggest that only moderate to large effect sizes are unlikely (95% Bayesian credible interval [− 0.550, 0.628]), and thus, between-group differences that have small to moderate effect sizes in either direction cannot be ruled out.

**Figure 4:**
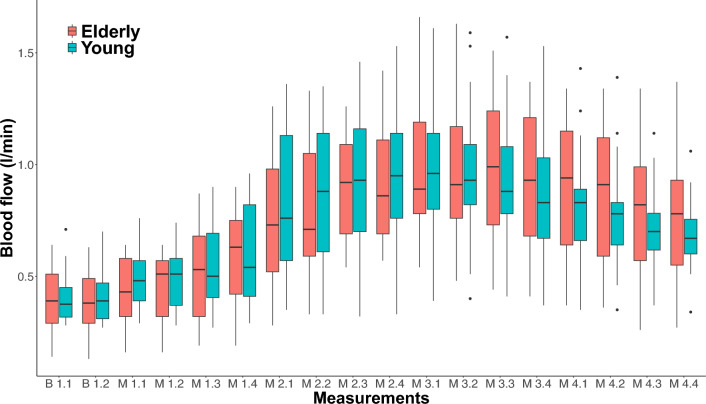
Box plot of postprandial superior mesenteric artery blood flow measurements in elderly and young participants. Boxes represent the interquartile range with horizontal lines indicating the median of the blood flow measurements (l/min) in each group at baseline (B1.x) before meal intake and each block of four consecutive blood flow measurements (M1.x–M4.x) interrupted by interspersed blood glucose measurements after meal intake. Whiskers represent the smallest and the largest values within 1.5 times the 25 and 75th percentiles, respectively. Dots represent extreme outlier values (i.e., values above 1.5 times the interquartile range). Blood flow measurements in each participant were repeated every 2-4 minutes depending on heart rate, why precise time for each measurement of blood flow differs between participants.

When comparing the smoothed postprandial increase in blood flow relative to baseline, there was still no significant difference between groups (*p* = 0.59, Table 1). Likewise, the maximal positive slope of the fitted curves, representing maximal rate of blood flow change, was not different in the elderly group relative to the slope in the young group (*p* = 0.92, Table 1). Furthermore, both the baseline blood flow levels, the postprandial maximal blood flow levels as well as the time to reach maximal blood flow did not differ between groups (Table 1). The box plot of the postprandial SMA blood flow measurements in elderly and young participants in Fig. 4 emphasizes the similarity in blood flow dynamics between groups.

**Figure 5:**
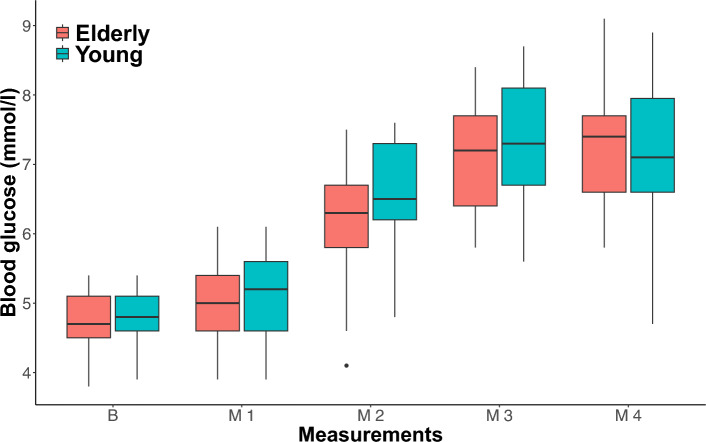
Box plot of blood glucose measurements in elderly and young participants. Boxes represent the interquartile range with horizontal lines indicating the median of the blood glucose measurements (mmol/l) in each group at baseline (B) before meal intake and blood glucose measurements (M1–M4) following each block of blood flow measurements after meal intake. Whiskers represent the smallest and the largest values within 1.5 times the 25 and 75th percentiles, respectively. Dots represent extreme outlier values (i.e., values above 1.5 times the interquartile range). Blood flow measurements in each participant were repeated every 2–4 minutes depending on heart rate, why precise time for each measurement of blood glucose differs between participants.

There was no significant between-group difference in blood glucose measurements between elderly and young subjects (Table 1). This is also illustrated by the box plot of blood glucose measurements in Fig. 5, that emphasizes that blood glucose dynamics are comparable between elderly and young participants. The time to reach maximum blood glucose level did not differ between groups (Table 1).

### Correlation analysis

Correlation analysis revealed a significant positive correlation using Pearson correlation coefficient (r) between blood flow at baseline and blood flow maximum when considering all participants (r = 0.564, *p* = 0.0005, Supplementary Fig. 1), showing that a higher pre-ingestion blood flow at baseline is associated with a higher post-ingestion blood flow maximum. R-square of this model was 0.318, which means that 31.8% of the variation in postprandial blood flow maximum is accounted for by inter-individual variations of the preprandial blood flow at baseline. This was the only correlation that survived a correction for multiple comparisons using Bonferroni correction. When considering the young and elderly group separately, this correlation was still found in both (Young group: r = 0.517, *p* = 0.03 and Elderly group: r = 0.617, *p* = 0.008), but was not present after Bonferroni correction.

### Post-hoc analysis

In a subgroup of participants, we observed a very early but transient increase in postprandial blood flow at the first measurement after meal intake. Therefore, we performed a post-hoc analysis, dividing the participants into a group with or without an early postprandial blood flow response. Demographic and clinical group data along with the statistical results of between-group comparisons are listed in Table 2.

**Table 2:** Demographic and clinical data of post-hoc analysis of subjects with and without early response in blood flow.

	Subjects without early response (n = 22)	Subjects with early response (n = 12)	P-value
Gender (male/female)	14/8	8/4	
Age (years)	43.9 ± 16.8	37 ± 20.5	0.33
Body mass index (kg/m^2^)	25 ± 3.4	24.5 ± 4.1	0.73
**GSRS (Items 1–9)**			
Total score	1.28 (1–1.67)	1.22 (1–1.43)	0.36
**Superior mesenteric artery blood flow (BF) (l/min)**			
Baseline BF	0.4 ± 0.12	0.4 ± 0.14	0.96
Postprandial maximal BF	1.19 ± 0.3	0.94 ± 0.2	0.005
Postprandial increase in BF	0.78 ± 0.24	0.54 ± 0.16	0.0009
Time to maximum (min)	30.1 (17.3–40)	27.5 (22.1–50.6)	0.68
**Blood glucose (BG) (mmol/l)**			
Baseline BG	4.7 ± 0.4	4.9 ± 0.4	0.19
Postprandial maximal BG	7.3 ± 0.8	7.7 ± 0.9	0.18
Postprandial increase in BG	2.6 ± 0.8	2.8 ± 0.8	0.39
Time to maximum (min)	54.7 ± 10.2	51.3 ± 10.8	0.39

Data given as mean ± SD or median (10% quantile**–**90% quantile). “Early response” is defined by immediate increase (> 2 × SD of baseline measurements) in the first measurement after meal intake and subsequent decrease in the second measurement.

At the group level, the absolute postprandial increase in SMA blood flow after food intake was significantly attenuated in the group with early response compared to the group without early response (*p* = 0.0009, Table 2 and Supplementary Figs. 2 and 3). Maximum blood flow levels were also found to be reduced in the group with early response (*p* = 0.005, Table 2). In contrast, the baseline levels as well as the time to reach maximal blood flow did not differ between groups. There was no significant between-group difference in blood glucose measurements (Table 2).

Again, we looked for correlations in the data, where we only found a significant positive correlation between blood flow at baseline and blood flow maximum in the group without early response (r = 0.662, *p* = 0.0008), showing that a higher pre-ingestion blood flow at baseline is associated with a higher post-ingestion blood flow maximum. However, this correlation was only borderline significant in the group with early response (r = 0.572, *p* = 0.052).

## Discussion

Using dynamic phase-contrast MRI, we found no evidence for age-related differences in functional regulation of gastrointestinal flow in response to food intake. Postprandial blood flow dynamics in SMA did not differ between healthy young and elderly subjects. This includes baseline blood flow levels, postprandial blood flow increase, postprandial maximal blood flow levels as well as the time to reach maximal blood flow. We did observe a noticeable inter-individual variation in postprandial intestinal blood flow increase, and approximately 30% of the variation in postprandial blood flow maximum can be explained by the preprandial blood flow. Regardless of age, some participants also showed a remarkable transient SMA blood flow increase immediately after meal intake.

To our knowledge, this is the first study comparing postprandial blood flow response in SMA after food intake between healthy young and elderly subjects. The only study investigating the age-related effect on SMA used infusion of intraduodenal glucose loads, which led to load-dependent rise in SMA blood flow in both young and older subjects with no difference in magnitude of change in SMA flow between them^21^. Interestingly, a study found that blood flow in SMA is significantly lower in children (5–9 years) than in adolescents (10–17 years) and is associated with body surface area^22^. Postprandial hyperemia has mainly been studied in the SMA using Doppler ultrasonography in healthy individuals, revealing wide-range variation among studies possibly due to the inherent difficulties in performing flow measurements using ultrasound^3^. While ultrasonography of the SMA has shown low interobserver variability in basic Doppler wave parameters^23^, different designs of these studies, including the composition of meal nutrients, might at least partly explain the observed wide-range variation.

A prominent finding was a large inter-individual variation in postprandial blood flow response in the SMA. This might be caused by factors not investigated in this study, for example caffeine. Other studies evaluated the effect of caffeine, commonly used for the treatment and prophylaxis of apnea of prematurity, on SMA blood flow velocities in preterm infants. While a single 10 mg/kg intravenous loading dose of caffeine did not cause a significant reduction in SMA blood flow velocities (BFV)^24^, BFV in SMA were significantly reduced after caffeine infusion at a loading dose of both 20 mg/kg and 25 mg/kg, which continued for at least 2 hours, but BFV in SMA were not significant affected by maintenance doses^25,26^. It is not possible to draw parallels from preterm infants to adults as in our study, however, it cannot be excluded that caffeine, which has a highly variable biological half-life, might have influenced the variability of SMA response. Also, smoking and eventual medicine intake, which did not lead to exclusion based on our criteria, might also have contributed to the considerable inter-individual variation which we observed.

Alterations in endothelial function in the aorta and mesenteric artery might also be a factor contributing to the large inter-individual variation in postprandial blood flow response. Studies indicate impaired vasomotor activities in the thoracic aorta and SMA in type II diabetic rats^27^, as well as abnormal SMA responses to endothelial stimulators in rats with kidney dysfunction^28^. Although the exclusion criteria of the current study aimed to minimize high-risk factors for endothelial dysfunction, vascular endothelial dysfunction could still contribute to the observed inter-individual variation. In addition, the participants' menstrual cycle or their use of contraception might have played a role in the large inter-individual variation in the postprandial blood flow response in the SMA. Sex and menstrual cycle phase have recently been shown to have minimal influence on hemodynamic responses in the celiac artery during dynamic moderate-intensity exercise in young healthy individuals^29^. Additionally, sex and the menstrual cycle have been shown to modulate endothelium-dependent flow-mediated dilatation of the brachial artery^30^. Whether sex and menstrual cycle phase influence gastrointestinal blood flow in response to food intake remains to be clarified in future studies.

Several mechanisms have been proposed to contribute to the regulation of gastrointestinal flow response to food intake including glucose absorption, motility, humoral and paracrine mechanisms, as well as extrinsic and intrinsic neural control of gastrointestinal perfusion.

A study in elderly subjects with and without critical illness measuring glucose absorption from the duodenum after glucose infusion by a postpyloric catheter reported a strong association between the nutrient-stimulated increase in SMA and total glucose absorption across all subjects^31^.

Also, it has been suggested that that there may be a relation between the rate of emptying of food from the stomach and the blood flow response. One study found a significant curvilinear relation between the superior mesenteric artery blood flow response and gastric emptying after intake of a high carbohydrate meal, but no relation after a high fat meal^32^. Recently, we could show that gastric emptying as measured with scintigraphy did not correlate with the postprandial blood flow response in the SMA neither in patients with Parkinsons Disease, nor in healthy age- and sex-matched controls^33^. Also, gastric emptying correlated neither with postprandial absolute blood flow increase nor with time to reach maximal blood flow in SMA. However, it is important to note, that gastric emptying may be related to changes in postprandial blood flow response in the arteries supplying the stomach and not necessarily the SMA, which supplies the gastrointestinal tract from lower part of the duodenum to the left colic flexure.

A variety of studies have implicated the intrinsic innervation of the gut by the enteric nervous system (ENS) in regulation of postprandial intestinal hyperemia^8,34–36^, while others cast doubt on the potential role of local intestinal nerves in postprandial regulation of intestinal blood flow^4,8,37^. Several studies suggest that extrinsic sympathetic and parasympathetic innervation of the gut may not play a significant role in regulation of postprandial hyperemia^4,9,37–39^. Conversely, in conscious dogs it was shown that postprandial celiac hyperemia, but not postprandial mesenteric hyperemia, is mediated by a vagal reflex^40,41^. In Parkinson’s disease the ENS, as well as the sympathetic and parasympathetic system are affected by α-synucleinopathy^42–44^. Therefore, the observed attenuated postprandial blood flow response in the SMA and the less prominent postprandial increase in blood glucose in patients with Parkinson’s disease^20^ might suggest that extrinsic and intrinsic neural inputs are implicated in regulation of postprandial hyperemia. However, the neurovascular regulation of postprandial intestinal hyperemia remains unclear, but most likely involves an interaction of several mediators and mechanisms^4,8,9^.

Disease may influence postprandial blood flow regulation in the SMA. In patients with Parkinson’s disease, we observed an attenuated postprandial blood flow response in the SMA compared to healthy individuals^20^. In critically ill patients aged over 65, the increase in SMA blood flow stimulated by intestinal glucose infusion was less compared with healthy subjects^31^. Also, patients with occlusive mesenteric ischemia displayed a smaller postprandial blood flow increase in the SMA after a meal intake^45^. The mechanisms underlying impaired regulation of gastrointestinal perfusion in response to food intake remain to be clarified. Various potential mechanisms need to be considered, including impaired glucose absorption, changes in motility, endothelial dysfunction, impaired humoral and paracrine function as well as impaired neural control of gastrointestinal perfusion.

Post-hoc analysis of our data showed a subgroup in both the young and elderly group, which had an “early response” with an immediate increase in the first measurement after meal intake and subsequent decrease in the second measurement. In the group with early response the absolute postprandial increase in SMA blood flow after food intake was significantly attenuated and maximum blood flow levels were also found to be reduced compared to the group without early response. This indicates that some subjects have an anticipatory rise in SMA blood flow, which subsequently results in an attenuated absolute increase and lower maximal blood flow levels.

The observed early response in this subgroup of both young and elderly subjects indicates that there might be anticipatory mechanism, which precedes the blood flow increase in each artery supplying the gastrointestinal tract as the digested chyme is exposed to the mucosal surface supplied by the artery. Someya et al. showed an increase in mean blood velocity in SMA within a minute after start of the meal, suggesting that the increase in SMA blood flow might precede the arrival of the chyme to the small intestine^3^. In contrast to our results, the magnitude of the initial increase in mean blood velocity in SMA was maintained for approximately 10 min and then markedly increased after 30 min. Interestingly, an old study from 1970 showed that in conscious dogs anticipation and ingestion of food were characterized by transient increases in heart rate, cardiac output and aortic blood pressure, whereas blood flow in SMA initially and transiently decreased by an average of 10%^40^. Heart rate, cardiac output, and aortic blood pressure returned to baseline levels after 10–30 min, whereas blood flow in SMA began to increase within 5–15 min of presentation of food. Another study from 1968 showed similar increases in heart rate, cardiac output, and aortic blood pressure during food intake in nonanesthetized dogs, while the ratio of blood flow in SMA to cardiac output decreased^46^. Interestingly, in this study absolute blood flow in SMA decreased during anticipation period of 45–60 s, increased during the 1 and 2 min of actual digestion and decreased again during the 3 min of actual digestion and one minute after the completion of ingestion.

The underlying mechanisms responsible for the observed early response in the subgroup of both young and elderly subjects are not clarified yet but might be mediated by activation of higher brain centers and the autonomic nerve system. It has been shown that sham-feeding (“chew-and-spit” technique), thinking about food as well as the sight and smell of food results in a significant increase in gastric secretion in healthy subjects^47^. Also, it has been shown that intrinsic gastric electrical activity can be altered by sham feeding^48^. However, a study demonstrated that chewing and taste increases blood velocity in the celiac artery but not the SMA, suggesting that taste of food might not play a role in anticipatory mechanism responsible for the observed early response in the SMA^49^. Future studies need to investigate these mechanisms, possibly by also measuring SMA blood flow during the anticipation period, where subject see and smell the test meal and during the actual ingestion of the test meal.

The current study has several limitations.

Participants did not undergo a detailed assessment of clinical state at the day of examination apart from GSRS and body mass index. Therefore, we do not know whether participants were current smokers or took any medicine not related to the exclusion criteria up to the examination day. Also, we do not know whether participants had caffeine intake before the 7 hours fast prior to MRI scanning.

In the current study, participants responded only to the first nine items of the GSRS about reflux, abdominal pain and indigestion, but not to the last 6 items, which assess diarrhea and constipation. Since SMA supplies the gastrointestinal tract from lower part of the duodenum to left colic flexure, a total score of all 15 GSRS items would have been more representative, and correlation analysis with subjective complaints related to lower gastrointestinal tract dysfunction would have been of interest. Also, participants were included independently of the presence or the degree of gastrointestinal symptoms. Future studies should investigate whether there are other factors that impact SMA blood flow response in a physiologically and/or clinically relevant manner. These factors may include constipation, participants' menstrual cycle, use of contraception, medication intake, smoking, and caffeine intake.

In the elderly group the majority of participants were middle-aged and only five were above 65 years old, therefore future studies might strive to include more elderly participants.

Body mass index was significantly higher in the elderly group compared to the young group in the current study. Correlation analysis showed no body mass index-related association to SMA blood flow and blood glucose values.

In this study, we exclusively focused on measuring blood flow in SMA, only interrupted by interspersed blood glucose measurements. This approach allowed for repeated blood flow measurements at short intervals. However, future studies should consider including blood pressure measurements between blood flow measurements and examining the ratio of blood flow in the SMA to cardiac output. This would serve the purpose of determining systemic vascular resistance in relation to mesenteric perfusion in response to food intake and, consequently, the relative blood flow distribution during the postprandial response.

In some participants, postprandial blood flow and serum glucose did not peak until towards the end of the time window covered by our measurements. The approximately 60-min postprandial measurement time was chosen based on Someya et al.'s summary of 10 studies on the human postprandial SMA blood flow response, which indicated peak flow between 5 and 60 min after a meal^3^. Considering our prior study involving elderly patients with Parkinson's disease in the OFF-state^20^, the maximal horizontal positioning time was estimated to be 60 min. A longer time window would have given the possibility to follow the parameters returning to baseline and to calculate the area under the curve (AUC). Future studies should therefore consider using a longer observation time than in the present study.

Using PC-MRI, we provide tentative evidence that postprandial blood flow dynamics in SMA in healthy young and elderly subjects do not substantially differ, indicating that age is without impact on vascular response in SMA as an indicator for regulation of mesenteric perfusion in response to food intake. Furthermore, we observed a considerable inter-individual variation in postprandial intestinal blood flow increase and approximately 30% of the variation in postprandial blood flow maximum can be explained by the preprandial blood flow at baseline. Future lines of research should identify the underlying mechanisms responsible for the large inter-individual variation, but also identify the underlying pathophysiological mechanisms responsible for impaired postprandial response in SMA in different diseases and explore its clinical significance.

## Methods

The methods, including the recruitment of participants, the experimental procedures, the use of the Gastrointestinal Symptom Rating Scale, the use of the standardized liquid test meal, and the statistical analysis, have been described previously by Siebner et al.^20,33^.

### Participants

Seventeen healthy young subjects aged < 30 years (range 19–28 years) and 17 healthy elderly subjects aged > 50 years (range 51–72 years) volunteered to participate in the present study. Apart from a single elderly individual, the data of the elderly subjects has already been described in our previously published study comparing postprandial changes in SMA blood flow in patients with Parkinson’s disease^20^. In compliance with the Helsinki Declaration, all participants provided written informed consent before their participation. The study was approved by the Regional Committee for Health Research Ethics of the Capital Region of Denmark (H-18054923) and the Danish Data Protection Agency. All methods were performed in accordance with relevant guidelines and regulations. Participants were recruited through advertising flyers posted at Copenhagen University Hospital – Amager and Hvidovre and through online advertisements posted on http://www.forsoegsperson.dk/, a Danish homepage for recruitment of study participants. Participants were included independently of the presence or the degree of gastrointestinal symptoms. Exclusion criteria were pregnancy or breastfeeding, diabetes mellitus, respiratory/cardiac/liver diseases, history of other neurologic or psychiatric disease, treatment with a pacemaker or other implanted electronic devices, and claustrophobia. None of the participants used laxatives on a daily basis.

### Experimental procedures

**Figure 6:**
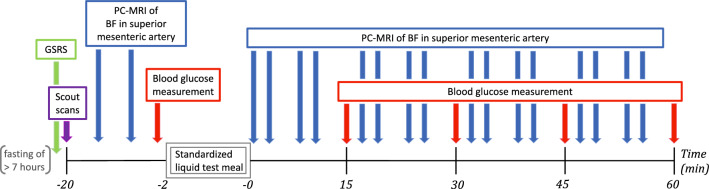
Experimental timeline for elderly and young subjects. Scout scans were performed to locate the superior mesenteric artery. The standardized liquid test meal consisted of 95 g Queal™ blended with 200 ml water. GSRS, Gastrointestinal Symptom Rating Scale (the first 9 items); PC-MRI, Phase-contrast magnetic resonance imaging; BF, Blood flow.

The experimental timeline and procedures are outlined in Fig. 6. Participants were instructed to fast for at least seven hours prior to MRI scanning. Postprandial increases in mesenteric blood flow were assessed using phase-contrast magnetic resonance imaging (PC-MRI) on a 1.5 T MRI scanner. While subjects were lying in the MRI scanner, blood glucose concentrations were measured from a drop of blood obtained by puncturing a fingertip, using a glucose meter (HemoCue^®^ Glucose 201 RT System, Denmark). All participants were scanned between 12:30 and 18:00.

Before PC-MRI, participants completed a questionnaire to assess subjective gastrointestinal symptoms. Participants were placed in the MRI scanner and the SMA was located with a scout scan. Two pre-meal baseline blood flow measurements in SMA were performed, followed by measurement of the baseline blood glucose level. Participants were given two minutes to ingest a standardized liquid test meal. They used a straw for meal intake while lying on the bed outside the scanner bore. Immediately after meal intake, we conducted blocks of four consecutive blood flow measurements in SMA using PC-MRI. Each block of blood flow measurements was followed by a new blood glucose measurement. Alternating blood flow and blood glucose measurement were repeated at least four times (Fig. 6).

### Gastrointestinal symptom rating scale

Participants completed the Gastrointestinal Symptom Rating Scale (GSRS) upon their arrival on the study day. Participants responded to the first nine items of the GSRS which assessed reflux, abdominal pain and indigestion^50,51^. Each item is rated on a 7-point Likert-type scale, ranging from 1 for the absence of symptoms to 7 for the most pronounced symptoms. The total score is then divided by the number of items to calculate the mean score. The recall period extended to the week preceding participation.

### Phase-contrast magnetic resonance imaging (PC-MRI)

The procedure and acquisition parameters related to PC-MRI have previously been described in detail by Siebner et al.^20^. MR imaging was conducted at Hvidovre Hospital’s Radiology Unit using a Siemens Avanto 1.5 T MRI Scanner (Siemens AG, Healthcare Sector, Erlangen, Germany). The participants were positioned supine in the scanner with MR compatible clothes. To achieve physiological blood flow conditions, participants were scanned at rest and were allowed to breathe freely. A 6-element Body MATRIX Coil (Siemens AG, Healthcare Sector, Erlangen, Germany) was placed on the participant’s abdomen. In addition, participants were equipped with hearing protection and an emergency call button. Electrocardiogram (ECG) for cardiac gating was obtained by placing MRI compatible electrodes on the participant’s chest and allowed for optimal retrospective reconstruction of PC-MRI scans. Subsequently, the participants were positioned with the abdomen in the isocenter of the scanner.

Firstly, a set of localizer sequences, including TRUFI (true fast imaging with steady-state free precession), HASTE (half-Fourier acquisition single-shot turbo spin-echo) and TOF (time of flight), were used to locate the SMA and to find the optimal slice position perpendicular to the vessel. The SMA was positioned centrally within the imaging slice, respectively, to optimize vessel identification during blood flow analysis. An appropriate velocity range for the participant in question was obtained by doing a VENC (velocity encoding) scout before performing PC-MRI.

Quantitative blood flow images were obtained by phase-contrast sequences using retrospective ECG-gated Cartesian two-dimensional cine fast low angle shots. Thirty cardiac phases were reconstructed per cardiac cycle. PC acquisition parameters were: repetition time/echo time = 49.45/3.37 ms, temporal resolution = approximately 15–33 ms depending on the heart rate, flip angle = 30 degrees, field of view = 120 × 120 mm, in-plane resolution = 0.896 × 0.625 mm, slice thickness = 5.0 mm, number of averages = 4, bandwidth = 521 Hz/Px. The proximal point where the SMA artery becomes perpendicular after arising from the aorta was used as a predetermined anatomical fixpoint for the flow measurements, so that the scans could be standardized.

We measured the blood flow dynamics in the SMA, which supplies the gastrointestinal tract from lower part of the duodenum to left colic flexure. During piloting, we tried to establish reliable blood flow measurements in the celiac artery, the left gastric artery and the common hepatic artery, but it turned out that these arteries could not be located and measured reliably, and we focused only on the postprandial response in SMA.

Our exclusive focus on the SMA enabled repeated blood flow measurements at short intervals. In the present study, blood flow measurements were repeated every 2–4 min (depending on heart rate) and were only interrupted by interspersed blood glucose measurements. Thus, our measurements reliably captured the temporal dynamics of the postprandial blood flow increase and systemic glucose uptake.

All flow data were subsequently analyzed by using CVI42 Version 5.6.5 (Software by Circle Cardiovascular Imaging BV, Amsterdam, The Netherlands). The region of interest was drawn manually by a single investigator (T.H.S.) in one of the 30 cardiac phase images by outlining the vessel wall. An automated contour detection method was then applied. Each phase image was carefully reviewed manually and corrected if necessary. Based on the area of the vessel and the mean blood velocity in the stipulated area, the blood flow was calculated for each of the 30 images as area times mean blood velocity. Subsequently, the mean arterial blood flow (l/min) during the cardiac cycle was calculated as the average value of these 30 individual values. This procedure was repeated for every PC-MRI measurement and for all participants. Since the results table produced by the CVI42 software does not provide vessel diameter or mean blood velocity as outcome variables, we cannot infer how much an increase in vessel diameter or an increase in mean blood velocity contributed to the postprandial blood flow increase in young or old individuals. Our measurements thus provide no information about how the relative contribution of these two physiological parameters to the postprandial blood flow increase changed with age.

### Standardized liquid test meal

Following baseline measurements, participants were given a standardized liquid test meal consisting of 95 g ready-made powdered shake mix Queal™ Steady Standard 5.0 (Queal BV, Rotterdam, The Netherlands) blended with 200 ml water at room temperature. QuealTM Steady Standard 5.0 is a ready-made powdered meal product, designed to provide full nutrition based on dietary standards with a ratio of macronutrients as recommended by the European Food Safety Authority and containing all 27 essential vitamins and minerals. 176 g of QuealTM Steady Standard 5.0 blended with 350 ml of water gives a complete meal of 700 kcal and contains 47% carbohydrates, 32% fats and 21% proteins.

During initial piloting of the present study the minimal amount of QuealTM Steady Standard 5.0, which still gave a significant increase in postprandial blood flow in the SMA, was found to be 95 g blended with 200 ml water.

95 g QuealTM Steady Standard 5.0 contains 398 kcal energy; 13,8 g fat of which 1,7 g saturated; 45,7 g carbohydrates of which 14,4 g sugars; 20 g protein; 6,7 g fibers and numerous vitamins and minerals.

### Post-hoc analysis

When visualizing the individual data, we observed a subgroup of young and elderly participants, who showed a very early but transient increase in postprandial blood flow at the first measurement after meal intake. This prompted us to systematically investigate this phenomenon. We defined as early response as an immediate increase in the first measurement after meal intake that exceeded twice the standard deviation of baseline measurements with a subsequent decrease in the second measurement. To characterize this early transient response further, we divided all participants post-hoc into a group with early response consisting of 12 subjects and a group without early response consisting of 22 subjects.

### Statistical analysis

Frequentist statistical analysis was conducted using R software (RStudio Inc., Version 2022.02.3). Based on Shapiro–Wilk test of normality, demographic and clinical group data in Table 1 and 2 are presented either as mean ± standard deviation or as median and 10%- & 90%-quantiles as appropriate. Wilcoxon rank-sum test was used to test for differences between groups when normal distribution could not be assumed, while unpaired two-sample t-test was used in the case of normal distribution. Statistical significance was accepted at *p* < 0.05. Correlations were determined using Pearson correlation and Spearman’s rank correlation as appropriate. The Bonferroni method was used to correct for multiple comparisons.

To infer on the absence of evidence, we perform Bayesian inference using JASP software (JASP, Version 0.18.3). This is only for one test where there the null hypothesis could not be rejected and inference on the null was theoretically interesting. We interpret the strength of evidence for Bayes factors using standard classifications (Jeffreys 1939, Kass & Raftery 1995).

The time at which the individual participant finished the intake of the standardized liquid test meal was set as t = 0 min. Serial blood flow (BF) data were expressed as percentage of individual baseline and calculated by (BF–Baseline BF)/Baseline BF * 100. To achieve robust, equidistant blood flow estimates, individual time series of blood flow measurements were smoothed using local polynomial regression fitting with a 25% smoothing span, and blood flow estimates were predicted at a sample rate of one sample per minute to extract the relative increase in blood flow, time to maximum and maximal slope.

### Supplementary Information

Supplementary Figures.

## Data Availability

The dataset is only pseudonymized and cannot be shared without a formal Data Processing Agreement or a formal approval by the Danish Data Protection Agency in order to meet the requirements of the GDPR and to ensure the protection of the rights of the data subject. Requests to access the datasets should be directed to Thomas H. Siebner, thomas.hartwig.siebner@regionh.dk.
