# Supplementary material for: No difference in postprandial mesenteric blood flow between healthy younger and elderly individuals
Source: Sci Rep. 2024 Apr 15;14:8689. doi: 10.1038/s41598-024-58111-w (PMC11018827; doi:10.1038/s41598-024-58111-w)
Supplement: Supplementary file 1 — Supplementary Figures. [file 41598_2024_58111_MOESM1_ESM.docx]

**Supplementary Figures**

**Supplementary Figure 1: Scatterplot of superior mesenteric artery blood flow at baseline and blood flow maximum in all participants.**

The red regression line represents the correlation between blood flow at baseline and blood flow maximum using Pearson correlation coefficient.

**Supplementary Figure 2: Postprandial superior mesenteric artery blood flow measurements in the group without early response and the group with early response.**

The absolute blood flow measurements are connected using linear interpolation. The black curve with shaded areas marks the mean and standard error for each group.

**Supplementary Figure 3: Boxplot of the absolute postprandial blood flow increase in superior mesenteric artery (SMA) in the group without early response and the group with early response.**

Boxes represent the interquartile range, which corresponds to the data between the 25th and 75th percentiles, with the bold horizontal line indicating the median of the increase in each group. The upper whisker extends to the largest value within 1.5 times the interquartile range above the 75th percentile, while the lower whisker extends to the smallest value within 1.5 times the interquartile range below the 25th percentile.
